# Cancer-associated fibroblasts-derived exosomal miR-3656 promotes the development and progression of esophageal squamous cell carcinoma via the ACAP2/PI3K-AKT signaling pathway

**DOI:** 10.7150/ijbs.62571

**Published:** 2021-08-27

**Authors:** Yuan Jin, Qilin Meng, Bihui Zhang, Chen Xie, Xue Chen, Baoqing Tian, Jiakang Wang, Tsung-Chieh Shih, Yibo Zhang, Jieqiong Cao, Yiqi Yang, Size Chen, Xinyuan Guan, Xiaojia Chen, An Hong

**Affiliations:** 1 Department of Cell Biology, College of Life Science and Technology, Jinan University, Guangzhou 510632, P. R. China; 2 National Engineering Research Center of Genetic Medicine, Guangzhou 510632, P. R. China; 3 Guangdong Province Key Laboratory of Bioengineering Medicine, Guangzhou 510632, P. R. China; 4 Guangdong Provincial biotechnology drug & Engineering Technology Research Center, Guangzhou 510632, P. R. China; 5 Shandong Cancer Hospital and Institute, Shandong First Medical University and Shandong Academy of Medical Sciences, Jinan, P. R. China; 6 Cancer Center of Guangzhou Medical University, Guangzhou 510090, P. R. China; 7 Department of Biochemistry and Molecular Medicine, University of California Davis, Sacramento, California, USA; 8 Oncology Department, The First Affiliated Hospital of Guangdong Pharmaceutical University, Guangzhou 510080, P. R. China; 9 Guangdong Provincial Engineering Research Center for Precise Therapy of Esophageal Cancer, Guangzhou 510080, P. R. China; 10 Department of Clinical Oncology, University of Hong Kong, Hong Kong, P. R. China

**Keywords:** Esophageal Squamous Cell Carcinoma (ESCC), Cancer-associated Fibroblast (CAF), Exosome, MicroRNA miR-3656, ACAP2

## Abstract

Esophageal squamous cell carcinoma (ESCC) is one of the most common gastrointestinal tumors, accounting for almost half a million deaths per year. Cancer-associated fibroblasts (CAFs) are the major constituent of the tumor microenvironment (TME) and dramatically impact ESCC progression. Recent evidence suggests that exosomes derived from CAFs are able to transmit regulating signals and promote ESCC development. In this study, we compared different the component ratios of miRNAs in exosomes secreted by CAFs in tumors and with those from normal fibroblasts (NFs) in precancerous tissues. The mRNA level of hsa-miR-3656 was significantly upregulated in the former exosomes. Subsequently, by comparing tumor cell development in vitro and in vivo, we found that the proliferation, migration and invasion capabilities of ESCC cells were significantly improved when miR-3656 was present. Further target gene analysis confirmed ACAP2 was a target gene regulated by miR-3656 and exhibited a negative regulatory effect on tumor proliferation. Additionally, the downregulation of ACAP2 triggered by exosomal-derived miR-3656 further promotes the activation of the PI3K/AKT and β-catenin signaling pathways and ultimately improves the growth of ESCC cells both in vitro and in xenograft models. These results may represent a potential therapeutic target for ESCC and provide a new basis for clinical treatment plans.

## Introduction

Esophageal squamous cell carcinoma (ESCC) has been one of the most common and deadliest cancers in recent decades and is the sixth leading cause of cancer-related death in the world 1-3. The number of ESCC cases has shown an increasing trend year by year in East Asia, South Africa, East Africa and Northern Europe 4, while less than 20% of advanced EC patients survive for 5 years 5, 6. Therefore, research on the carcinogenic mechanism of ESCC and the determination of therapeutic targets are beneficial for optimizing the treatment plan for this deadly disease.

The tumor microenvironment (TME) is the internal environmental system composed of tumor cells, tumor-associated cells and cytokines. Tumor cells capture surrounding cells, resulting in depletion of immune-related cells or induction of fibroblasts to produce regulatory signals that promote tumor development 7-10. Tumor-associated fibroblasts (CAFs) can release stromal cell-derived factors and proangiogenic factors to promote tumor cell growth and tumor angiogenesis 11-15. Communication between ESCC cells and CAFs is accompanied by this two-way, multichannel material exchange. With in-depth research on non-coding RNAs and the transfer of macromolecular substances between cells, it has been suggested that CAFs communicate with tumor cells to impact tumor development via microRNAs in exosomes as vehicles 16-19.

Exosomes are extracellular membrane vesicles enveloping different ratios of DNA, RNA and protein components with diameters of 40-120 nm 20-24. Numerous reports indicate that exosomes are excreted by various cells and are naturally present in body fluids, including blood, saliva, urine, cerebrospinal fluid and milk, representing a ubiquitous means of communication between cells. Exosomes are characterized by their specifically associated antigens, such as Alix (ALG-2-interacting protein X), integrins, Rab GTPases, tetraspanins (CD63, CD9, CD81 and CD82) and TSG101 (tumor susceptibility gene 101) 25-27. Since the molecules in exosomes are protected from rapid degradation, exosomes are involved in cell-cell communication for signal transduction or horizontal transfer of information by diffusion or systemic transport to distant anatomical locations 28-33. MicroRNAs (miRNAs) are a type of non-coding single-stranded RNA molecule that can bind to the 3'-untranslated region (UTR) of target mRNA through base pairing, thereby negatively regulating gene expression at the posttranscriptional level by reducing the mRNA lifespan in the cytoplasm. Recently, it has been reported that during the development of ESCC, the level of some miRNAs is obviously changed in tumors and tumor-related cells^34^-^36^, but the mechanism of action has not been fully elucidated.

In this study, we used high-throughput miRNA sequencing to compare the different components and ratios of miRNAs in the CAF-derived exosomes (CDEs) secreted by CAFs cells in tumors and the NBEs from normal fibroblasts (NFs) in precancerous tissues, and we found that CDEs induced higher rates of proliferation and migration of ESCC cells than normal-fibroblast-derived exosomes (NDEs). Further studies revealed that the level of miR-3656 was significantly higher in CDEs than in NDEs. In vitro assays showed that miR-3656 promoted the proliferation, invasion and migration of ESCC via exosomes by targeting ACAP2 to activate the PI3K/AKT and β-catenin signaling pathways. Additionally, in vivo assays showed that miR-3656 promoted the growth of ESCC xenografts (PDXs). Our findings indicated that miR-3656 from CDEs promotes ESCC cell growth and PDX development, suggesting that miR-3656 may be a new signal from CAFs promoting the development of ESCC.

## Materials and Methods

### Cell Culture

Samples of tumor tissue and precancerous tissue were collected from five patients who did not receive any preoperative chemotherapy or radiotherapy enrolled in the Cancer Institute and Hospital, Guangzhou Medical University (Guangzhou, China). CAFs and NFs were separately derived from tumor tissues and precancerous tissues. Patient-derived ESCC cell lines, human ESCC cell lines (EC-18 and KYSE30) and embryonic kidney cell line 293 (HEK293) were provided by Professor Guan from the Department of Clinical Oncology, The University of Hong Kong. The EC-18, KYSE30 and HEK293 cell line were STR qualified by DSMZ (Braunschweig, Germany). The cells were cultured in DMEM (HyClone, Thermo Fisher, USA) containing 10% FBS (HyClone, Thermo Fisher, USA) in a 5% CO_2_, 37 ℃ incubator. Cells were confirmed to be mycoplasma-free with a Mycoplasma Detection Kit (Southern Biotech, USA). In terms of expression intervention, the miR-3656, siRNA of ACAP2 and control RNAs were chemosynthetic and purified with HPLC (GenScript, China) according to the reported sequence. Lentiviruses inserted with miR-3656 sequences (Lenti-miR-3656), ACAP2 sequences (Lenti-ACAP2), nonsense RNAi control sequences (Lenti-mimic) and no exogenous sequence (Lenti-Vector) were constructed and purified to 1 × 10^9^ CFU by HanBio, China. The operation of lentivirus transfection of ESCC cells and the operation of screening by Purimycin were performed according to the supplier's instructions.

### Isolation and Quantification of Exosomes

After culturing CAFs and NFs for 72 hr, the supernatants were collected and subjected to gradient centrifugation (300 g, 2000 g and 12000 g, respectively, for 30 minutes) followed by filtering through a 10K Centricon Plus-70 ultrafiltration tube (Millipore, USA) to remove cell debris and impurities. The remaining supernatant was purified by an EXO Quick-TC exosome isolation kit (EXOTC50A, System Biosciences, USA) according to the instructions. The protein concentration of the obtained exosome suspension was determined with a BCA Protein Assay Kit (Biyuntian, China), and the newly prepared exosomes were subjected to transmission electron microscopy (TEM) observation with TEM-2000 EX II (JEOL, Tokyo, Japan) as previously described 37. The size distribution of exosomes was determined by a Zetasizer Nano ZS90 (Malvern, UK) light scattering size potentiometer according to a previous report 38. Cell proliferation was assessed with a Cell Counting Kit-8 (Dojindo, Japan) according to the user manual with 5 repeats, while absorbance at 450 nm was recorded using a microplate spectrophotometer (NanoDrop 3300, Thermo, US).

### Cell migration, colony formation and wound healing assays

A cell migration assay was performed to investigate cell migration using a Transwell insert that contained polycarbonate filters with 8-μm pores (Corning, USA) according to a previous report 39. Migrated cells were visualized and observed under a microscope (IX71, Olympus, Japan). Rates of cell migration were quantitated by counting the cell number attached to the lower membrane surface in 10 random fields. Colony formation assays were conducted according to previous research 40. The images were detected under a microscope (IX71, Olympus, Japan), and colonies were counted using NIH ImageJ. Wound healing assays were carried out as previously described 41. The scratched areas were recorded under a microscope (IX71, Olympus, Japan) at 0 or 24 hr, and distances of cell migration were analyzed using NIH ImageJ.

### Fluorescent immunostaining

Luciferase reporter vectors encoding the wild-type 3' untranslated regions or mutant 3' UTRs of APAC2 were constructed. The luciferase activities were measured by the Dual Luciferase Reporter Assay Kit (Beyotime Biotechnology, Shanghai, China) according to the manufacturer's instructions.

### MicroRNA sequencing

The total RNA of exosomes was prepared for RNA sequencing as mentioned previously^42^. In simple, the total RNA of tissue samples was extracted by Trizol method after a frozen-grinding. The quality of RNA samples was inspected by UV absorbance and nucleic acid electrophoresis. RNA Libraries were prepared with Truseq Small RNA Sample Preparation Kits (illumina, USA) and purified for deep sequence on the illumina Hiseq 2500. The clean reads were determined, and differential expression profiles were analyzed by BetterWays Inc., China. The target mapping and functional annotation of microRNAs were completed through querying TargetScan 3.1 Database (www.targetscan.org) and MicroRNA Target Prediction Database (www.mirdb.org).

### Quantitative real-time PCR

Total RNA was isolated from cells using RNAiso Plus (Takara, Japan), and cDNA was synthesized using a first-strand reverse transcription kit (Takara, Japan) according to the manufacturers' instructions. Total RNA of exosomes was isolated using SeraMir Exosome RNA Amplification (RA808A-1, System Biosciences, USA). cDNA was synthesized from total RNA using the miDETECT A TrackTM miRNA qRT-PCR Starter Kit (RiboBio, Guangzhou, China). RT-qPCR was performed on a Biometra RealTime PCR Detection System with SYBR Green Dye Mix (TaKaRa, Japan). U6 snRNA was used as an endogenous control to normalize miRNA expression in cells, and cel-miR-39 was used to normalize miRNA expression in exosomes between the samples. The primers for miRNAs were synthesized by RiboBio, China. The sequences were presented in Supplementary Table 1. Quantitative PCR was performed using the IQ5 Multicolor Real-Time PCR Detection System (Bio-Rad, Berkeley, CA, USA). The primers for RNU6 and cel-miR-39 were purchased from RiboBio Co., Ltd, China. Relative expression levels of miRNA and mRNA were calculated by the ^ΔΔ^Ct method.

### Western blot analysis

Primary antibodies against AKT (#4685S), p-AKT (#4060T), β-catenin (#8480T), CD63 (#55051S), CD81 (#56039) and CD9 (#13403) were purchased from Cell Signaling Technology, USA. The HRP-labeled anti-rabbit IgG monoclonal antibody (#D110065, Sangon Biotech, China) was used as the secondary antibody, and the fluorescent signal was developed with ECL substrate (#SW2010, Solarbio, China). The chemiluminescent signals were captured using a Bio-Rad ChemiDoc XRS system (Bio-Rad, USA).

### Animal experiments and welfare

All animal experiments were conducted following protocols approved by the Institute of Laboratory Animal Science, Jinan University, China. Four- to six-week-old female Balb/c mice were used to examine allograft tumor growth. A total of 1 × 10^7^ cells of the cell lines EC18-3656(OE), KYSE30-3656(OE), which overexpressed miR-3656, and the unmodified control cell lines EC18-NC and KYSE30-NC were respectively injected subcutaneously into mice. Animals were randomly divided into six groups. Tumor growth in nude mice was monitored over a 4-week period. The tumor volume was calculated by the formula V = 0.5 × L × W^2^. Tumors were weighed after harvesting to compile tumor growth curves.

### Statistical analysis

The experimental data were analyzed with GraphPad Prism 9 software. The results are presented as the mean ± standard deviation (SD). The significance of the differences was statistically analyzed by a two-tailed Student's t-test or one-way analysis of variance. The DFS curves were assessed by the Kaplan-Meier method based on the data set deposited in a public database (http://kmplot.com/analysis/). Protein expression data in clinical cases were retrieved from the Oncomine database (https://www.oncomine.com/). Identification of downstream targets and analysis of target-involved signaling pathways were performed with the Ingenuity Pathways Analysis (IPA) program. The Venn diagram was analyzed on the website (https://bioinfogp.cnb.csic.es/tools/venny/index.html). All measurements were conducted at least three times for statistical repetition unless otherwise stated. Differences were considered significant and labeled as * when p<0.05; **, p < 0.01 and ***, p <0.001.

## Results

### CDEs promote ESCC cell progression in vitro

**Figure 1:**
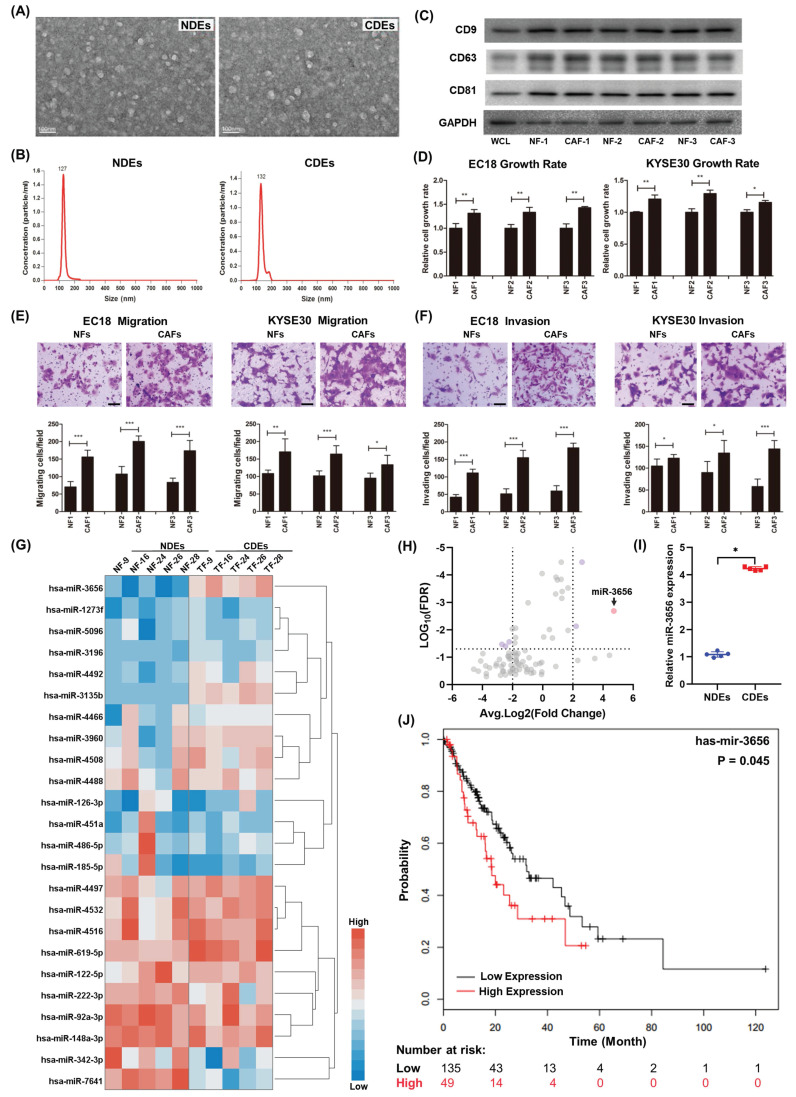
** miR-3656 upregulates CDEs to enhance ESCC development. (A)** Electron microscopy images (bar=100 nm) and **(B)** nanoparticle particle size distribution of CDEs and NDEs.** (C)** Western blot analyses of the exosomal protein markers in CDEs and NDEs from three different patients. Whole cell lysis was used as an input control; GAPDH was used as a loading control.** (D)** Plots showing the relative growth rates of EC18 and KYSE30 cells treated with exosomes for 48 hr. **(E)** Representative images (top) and quantification (down) of migratory or **(F)** invasive EC18 or KYSE30 cells per field after treatment with exosomes for 48 hr. Bars in plots represent mean ± SD. Scale bars in images equal to 50 µm. p > 0.05; *, p ≤ 0.05; **, p ≤ 0.01; and ***, p ≤ 0.001. **(G)** Plots showing the relative levels of a list of miRNAs in CDEs and NDEs prepared from 5 patients. **(H)** Distribution map drawn by expression level and false discovery rate. **(I)** The relative concentration comparison of miR-3656 in NDEs and CDEs as measured by RT-qPCR. **(J)** Kaplan-Meier analysis showing that individuals with high miR-3656 expression presented shorter DFS in 184 EC patients.

The diameters of exosomes separated in NFs and CAFs were distributed from 40-120 nm, and the difference in shape under TME and particle size between the two was not obvious (Figure 1A, B). The purified exosomes contained higher concentrations of characteristic markers (CD9, CD63, and CD81) than the whole-cell composition (WCL) (Figure 1C). To determine the effect of CDEs and NDEs on ESCC progression in vitro, we treated EC18 and KYSE30 cells with 20 ng/ml individual exosomes. Cells treated with CDEs had significantly higher rates of growth, migration and invasion than those treated with the same concentration of NDEs (Figure 1D-F). This suggests that CDEs can better promote the proliferation of tumor cells in vitro, which is different from NDEs.

### MiR-3656 is enriched in CDEs and negatively correlated with disease-free survival (DFS) in EC patients

Previous studies show that cancer cells secrete exosomes in large amounts to transfer cancer-associated signaling molecules to their surrounding cells 43, 44. Additionally, posttranscriptional gene expression in recipient cells can be regulated by miRNAs contained in exosomes. We speculated that there may be microRNAs in CDEs involved in this modulation. For this, we analyzed the microRNA profiles of CDEs and NDEs from five patients without chemotherapy and radiotherapy histories. The results showed that the level of a series of miRNAs changed significantly and that there were group-related differences (Figure 1G and Supplementary Table 2). Among these miRNAs, miR-3656 showed a significant upregulation in CDEs compared to NDEs (Figure 1H, I). Moreover, the expression levels of miR-3656 were negatively correlated with the survival probability of ESCC patients according to UALCAN analysis (Figure 1J).

### MiR-3656 promotes ESCC cells in vitro

**Figure 2:**
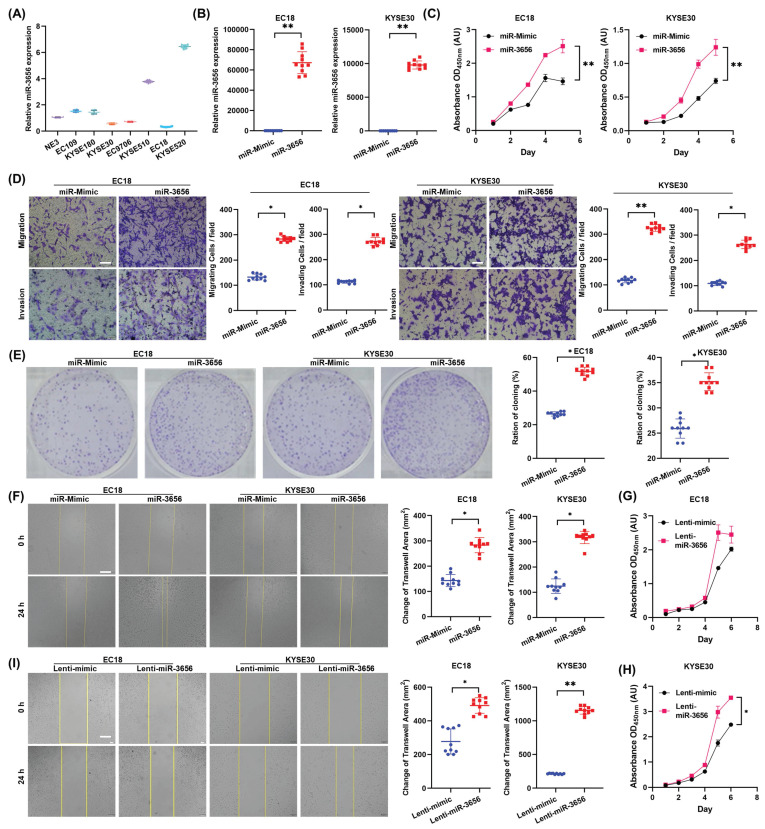
** miR-3656 impacts the proliferation and metastasis of ESCC cells in vitro. (A)** Plots showing the relative levels measured by RT-qPCR of miR-3656 among a panel of untreated ECSS cells and **(B)** between EC18 or KYSE30 cells treated with chemically synthesized miR-3656 and negative control miRNA without homology. **(C)** Plots showing the impacts of miR-3656 on the growth rates of EC18 and KYSE30 cells determined by CCK-8 assay. **(D)** Representative images and quantification showing the impacts of miR-3656 mimics on the rates of migration and invasion of EC18 and KYSE30 cells determined by Transwell assays (bar=50 µm), **(E)** colony formation assays and **(F)** wound healing assays (bar=250 µm). **(G-I)** Similar promotive roles were authentically repeated in lentivirus-transfected ESCC cells stably overexpressing miR-3656 (bar=250 µm).

To determine whether elevated expression of miR-3656 in CDEs promotes ESCC progression, we measured the expression of miR-3656 in human ESCC cell lines and selected two cell lines with low expression levels of miR-3656, EC18 and KYSE30 (Figure 2A). Then, we transfected EC18 and KYSE30 cells with chemosynthetic miR-3656 and mimic miRNA. The level of miR-3656 in both cell lines was upregulated according to RT-qPCR (Figure 2B). The proliferation rates of EC18 and KYSE30 cells were significantly increased (Figure 2C), while the migration activity, invasion activity and clone formation capacity appeared to be consistent with proliferation (Figure 2D, E). The wound-healing assay further confirmed that the migration ability of cells was markedly improved after transfection with miR-3656 (Figure 2F). Similar promotive roles were confirmed in lentivirus-transfected ESCC cells stably overexpressing miR-3656 (Figure 2G-I). These results suggest that miR-3656 expressed endogenously or delivered through exogenous vehicles can promote ESCC cell development in vitro.

### MiR-3656 promotes the development of ESCC cells in vivo

**Figure 3:**
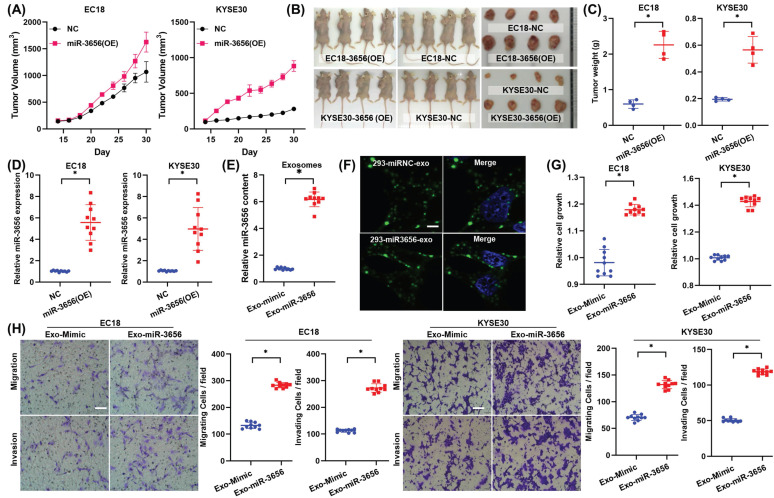
** miR-3656 but exosomal vehicle or CAFs enhance the rates of ESCC tumor growth in vivo. (A)** Plots showing tumor volume within 30 days post cell injection. **(B)** The appearance of tumor mass in transplanted tumor mice and the tumor tissue after dissection. **(C)** Plots showing tumor weights after harvesting. **(D)** Comparison of the expression levels of miR-3656 in fast-growing tumor tissue and normal tumor tissue. **(E)** The expression of miR-3656 in exosomes derived from the HEK293 cell line overexpressing miR-3656 constructed by gene editing was higher than that in the control group.** (F)** Representative images showing that exosomes labeled with the exosome-specific marker PKH67 (green) entered and were contained in ESCC cells.** (G-H)** The growth rate, invasion intensity and migration activity of ESCC cells were further compared, and ESCC cell lines treated with HEK293-derived exosomes overexpressing miR-3656 had an increased proliferation ability.

To explore whether miR-3656 has similar tumor-promoting effects in vivo, we established a mouse tumor model by subcutaneously inoculating miR-3656-expressing cells (miR-3656(OE)) and normal cancer cell lines (NC). The average weights and sizes of tumor nodules in miR-3656(OE) ESCC cells were higher than those in the control group (Figure 3A-C). The expression levels of miR-3656 in fast-growing tumor tissue of the miR-3656(OE) group were significantly higher than those of the NC group (Figure 3D). These results demonstrated that the cell lines with miR-3656 overexpression had accelerated tumor formation and enhanced tumor development in vivo. This indicated that the expression of miR-3656 in tumor cells promotes tumor development.

### The activity of miR-3656 relies on neither exosome vehicle nor CAFs

To verify whether the proliferation induction of miR-3656 is related to the source of exosome vehicles or CAFs, we constructed and isolated heterologous exosomes in HEK293 cells. The HEK293 cell line was transfected with lentiviral vector containing pre-miR-3656 or negative control vector containing scrambled shRNA, and the exosomes of each group (Exo-miR-3656 and Exo-mimic) were purified with the protocol mentioned above. The higher concentration of miR-3656 in Exo-miR-3656 exosomes was confirmed using RT-qPCR (Figure 3E). Equal amounts of exosomes were stained with PKH67 and co-incubated with ESCC cells (0 hr). Microscopic observation showed the outer green fluorescence of PKH67 concentrated on the surface of the cell membrane and inside the cell, indicating that ESCC cells can normally ingest HEK293-derived exosomes (Figure 3F). After cultivation for 48 hr, the growth rate, invasion intensity and migration activity of ESCC cells were further compared. The results showed that as Exo-miR-3656 was derived from HEK293 cells, the development of ESCC cells in vitro was significantly improved (Figure 3G-H). Combined with the results of the previous section, it is suggested that the cancer-promoting role of miR-3656 does not depend on exosome vehicles or CAFs.

### ACAP2 is the key target of miR-3656 in promoting ESCC

**Figure 4:**
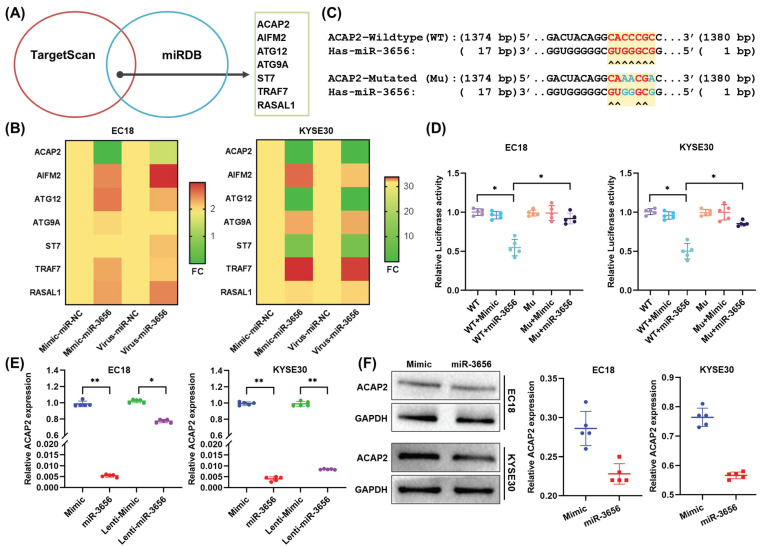
** miR-3656 directly targets ACAP2 and represses its expression. (A)** Venn diagram showing the miR-3656 target genes associated with tumor progression. **(B)** Heat map showing the expression changes of 7 candidate genes. **(C)** A diagram showing the 3′-UTR fragment of the human ACAP2 gene containing wild-type or mutated miR-3656 in the binding sequence of luciferase reporter vectors. **(D)** The relative luciferase activities in EC18 and KYSE30 cells expressing wild-type (WT) or mutant miR-3656 binding site (MU) untreated or treated with negative control (Mimic) or miR-3656 mimics (miR3656). **(E)** Comparison of the relative expression level of ACAP2 mRNA between transient (exogenous) transfection and lentivirus stable (endogenous production) cell lines. **(F)** The protein levels of ACAP2 in EC18 and KYSE30 cells treated with mimic or miR-3656.

Since microRNAs can perform multitarget gene expression regulation, to reveal the exact downstream target proteins related to tumorigenesis and development, we employed the TargetScan and miRDB databases to predict a set of common target genes of miR-3656. Seven genes were verified to be direct targets of miR-3656 and annotated as tumor progression promoters (Figure 4A). In the following step, the mRNA concentration of these 7 genes was determined using RT-qPCR, and we found that ACAP2 was most affected (Figure 4B). Moreover, to verify whether ACAP2 plays a crucial role, we analyzed the binding site of miR-3656 on the ACAP2 mRNA sequence and cloned the 3' UTR fragment of ACAP2 containing a miR-3656 binding site (WT-ACAP2) and mutant fragments (MUT-ACAP2) into luciferase reporter vectors (Figure 4C). The results revealed that miR-3656 significantly reduced luciferase activity in both EC18 and KYSE30 cells expressing WT-ACAP2 but not MUT-ACAP2 (Figure 4D), indicating that miR-3656 can regulate the expression of ACAP2 in ESCC cells. In addition, the ACAP2 mRNA and protein levels in both EC18 and KYSE30 cells were substantially reduced upon miR-3656 overexpression (Figure 6E, F). These results prompted us to conclude that ACAP2 is the key downstream link of miR-3656 and has a tumor-promoting function.

**Figure 5:**
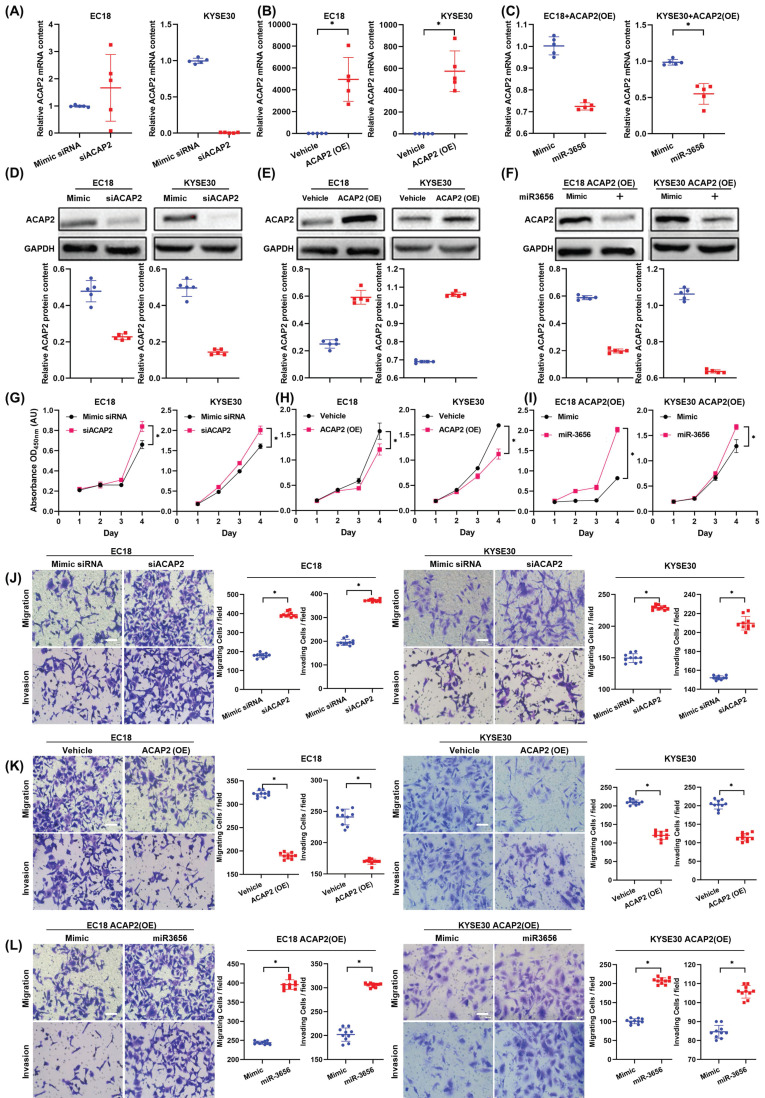
** ACAP2 mediates the impacts of miR-3656 on the proliferation and metastasis of ESCC cells.** Respectively, reducing ACAP2 expression with siRNA, overexpressing ACAP2 expression by lentiviral vectors pLenti-ACAP2, and repression of ACAP2 expression in overexpressing cell lines by transfecting miR-3656. **(A-C)** The mRNA level of each group was determined with RT-qPCR and** (D-F)** the ACAP2 protein level in each group were observed via Western Blot. After the level of ACAP2 was intervened, **(G-I)** the proliferation ability, **(J-L)** migration and invasion intensity of ESCC cells showed a negatively correlation with the level of ACAP2.

To test this hypothesis, we first determined the relationship between changes in ACAP2 expression and tumor development in the absence of miR-3656 by transfecting equivalent siACAP2 siRNA into ESCC cells. The results show that the decrease in ACAP2 mRNA level caused by siACAP2 (Figure 5A) can reduce the expression of ACAP2 protein in cells (Figure 5D) and significantly promote tumor proliferation, migration and invasion (Figure 5G, J). After that, we used lentiviral vectors to construct ESCC cell lines highly expressing ACAP2. The data comparison showed that with the high expression of ACAP2 (Figure 5B, E), the proliferation ability of ESCC cells decreased, and migration and invasion ability were simultaneously weakened (Figure 5H, K). Finally, we applied miR-3656 and mimic RNA intervention in ESCC cell models with high expression of ACAP2. The results show that the presence of miR-3656 can significantly reduce the mRNA and protein levels of ACAP2 (Figure 5C, F), which confirmed our previous result, and that miR-3656 recovered the proliferation, migration and invasion capabilities of ESCC cell lines that were suppressed by high expression of ACAP2 (Figure 5I, L). Based on the above comparison results, it is concluded that ACAP2 is the key target of miR-3656 during its promotion of ESCC cell development.

### The miR-3656/ACAP2 axis impacts both the PI3K/AKT and classical Wnt signaling pathways

**Figure 6:**
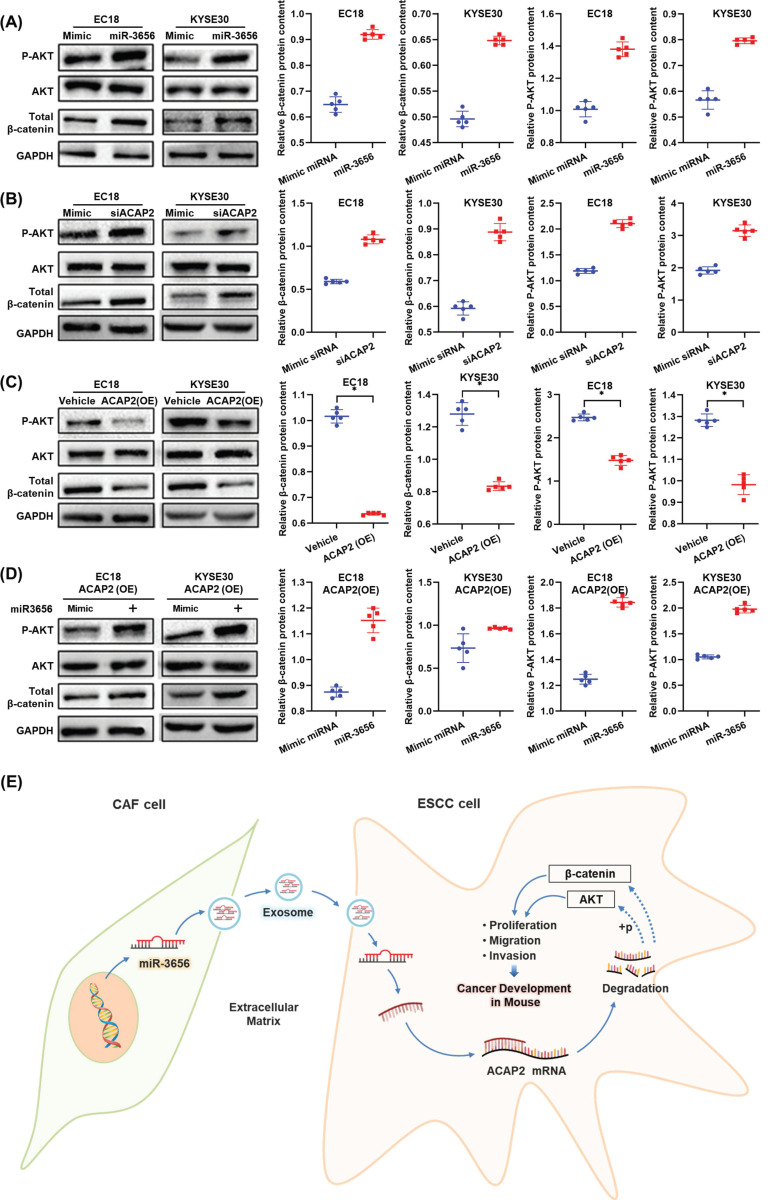
** The miR-3656/ACAP2 axis impacts both PI3K/AKT and classical Wnt signaling pathway cascades in ESCC cells. (A-D)** Representative immunoblots and quantitation of relative levels of phosphorylated AKT (p-AKT) and total AKT and β-catenin in EC18 and KYSE30 cells treated with miR-3656, ACAP2-targeted siRNA, ACAP2 overexpression and expression reversion by miR-3656 transfection after overexpression.** (E)** Schematic diagram of the inference based on existing data.

Based on the results of protein expression microarrays (data not shown), we observed high activation of the PI3K/AKT and Wnt signaling pathways under the action of miR-3656. Thus, with the help of the cell model used in the previous section, we continued to analyze the expression and phosphorylation of key node proteins in these two pathways. The results showed that the levels of both β-catenin and phosphorylated AKT were elevated when ACAP2 expression was suppressed either by effective siRNA or miR-3656, and the positive trend was reversed when the expression of ACAP2 was knocked in (Figure 6A-D). This suggested that the activation of both the PI3K/AKT and Wnt signaling pathways was consistent with the level of ACAP2, which was simultaneously regulated by miR-3656. In summary, based on the above data, we conclude that the exosome-carried miR-3656/ACAP2 axis may be responsible for CAFs promoting ESCC development (Figure 6E).

## Discussion

Recent studies have revealed that CAFs stimulate tumor growth by secreting various molecules to prepare a suitable microenvironment for tumor proliferation, angiogenesis, and invasion^45^, ^46^. An increasing number of reports have shown the importance of exosomes in the mediation of cancer progression 47. However, the mechanism underlying how CAFs impact ESCC progression has not been fully clarified. It was shown that the appearance of circulating miRNAs suggests physiological and pathological changes in patients and may potentially be used as biomarkers for the noninvasive screening of early-stage cancers 34, 35, 48-50. Similarly, circulating exosomal miRNAs originating from the TME have been found to be associated with therapy resistance or the progression of cancers 29, 51-56. Exosomal miRNAs have also been shown to be potential biomarkers for diagnoses in ESCC 57. However, it remains unclear whether specific miRNA-encapsulated CDEs potentially play a role in ESCC. To determine the molecular mechanism of CDEs, we isolated exosomes from five pairs of tumor materials and adjacent tissue excisions from patients without clinical intervention. With the help of high-throughput sequencing analysis, we identified microRNAs with obvious changes of expression level. By conducting a series of experiments, we demonstrated that miR-3656 was significantly upregulated in CDEs; exosomes derived from CAFs and NFs could be transferred to ESCC cells; and miR-3656 could facilitate ESCC cell proliferation and migration by targeting ACAP2. Analyses of the Kaplan-Meier datasets revealed that miR-3656 expression was positively correlated with poor DFS of EC patients. These findings suggested that miR-3656-containing exosomes derived from CAFs contribute to the malignant progression of ESCC.

Furthermore, the interruption of the PI3K-AKT pathway affects a wide range of tumor cellular functions, including cell proliferation, metabolism, cell apoptosis and survival^58^. A large number of downstream effectors in the ACAP2-PI3K-AKT-mediated oncogenic axis were previously reported. Sullivan et al. reported that ACAP2 inactivated AKT downstream signaling, and the inactivation or downregulation of AKT downstream signaling triggered a proapoptotic function in cancer cells that may contribute to the development of cancers^59^. We also found that the expression levels of ACAP2 were significantly downregulated in some esophageal cancers, and a similar trend was observed in both leukemias and lymphomas based on the analysis of the Oncomine database. These results suggest that ACAP2 is one of the key molecules that regulate tumor cell proliferation by negatively affecting the PI3K-AKT pathway in ESCC. In addition, current reports suggest that ACAP2 helps the formation of tumor-promoting exosomes. Jackson et al. determined that ACAP2 was recruited to platelet-derived growth factor (PDGF)-induced dorsal membrane ruffles in mouse fibroblasts, and its overexpression inhibited the formation of such ruffles^60^. We observed that in HEK293 cells whose ACAP2 expression was restricted by miR-3656, both the number of exosomes and the amounts of miR-3656 in exosomes were increased correspondingly. This indicates that the downregulated expression of ACAP2 may consequently promote exocytosis and the expression of exosomes. Taken together, previous reports and our results indicate that ACAP2 plays multiple regulatory roles in miR-3656 promoting the development of ESCC. In summary, our results indicated that the miR-3656/ACAP2 axis promotes cell migration and invasion through the AKT and β-catenin signaling pathways and might represent a novel target in ESCC therapy.

Interestingly, we found that miR-3656 does not depend on exosome vehicles or CAFs because regardless of whether it is derived from tumor tissue-derived exosomes, a high abundance of exocrine miR-3656 secreted by genetically modified HEK293 cells, or through transfection of chemically synthesized miR-3656 or a vector inducing miR-3656 overexpression, the proliferation of tumor cells can always be reinforced by the presence of miR-3656. This phenomenon is noteworthy because miR-3656 is not unique to ESCC or even to CAFs but may exist in a wider range to play a role in proliferation regulation. This speculation offers a direction for our future research.

## Supplementary Material

Supplementary tables.
