# Supplementary material for: Cancer-associated fibroblasts-derived exosomal miR-3656 promotes the development and progression of esophageal squamous cell carcinoma via the ACAP2/PI3K-AKT signaling pathway
Source: Int J Biol Sci. 2021 Aug 27;17(14):3689–701. doi: 10.7150/ijbs.62571 (PMC8495391; doi:10.7150/ijbs.62571)
Supplement: Supplementary file 1 — Supplementary tables. [file ijbsv17p3689s1.pdf]

Table S1 | A list of nucleotide sequences of primers and miR-3656

Gene name	Primer (5'-3')
ACAP2	F：AAGATGACTGTGGATTTCGAGGA<br>R：TGGTCAAACTTGTCTCAACGAC
AIFM2	F：AGACAGGGTTCGCCAAAAAGA<br>R：CAGGTCTATCCCCACTACTAGC
ATG12	F：CTGCTGGCGACACCAAGAAA<br>R：CGTGTTCGCTCTACTGCCC
ATG9A	F：CTGCCCTTCCGTATTGCAC<br>R：CTCACGTTTGTGGATGCAGAT
C1QTNF1	F：GTGCCCCAGATCAACATCACT<br>R：CCGTGTCGAAGATCACCGTC
RASAL1	F：CAGCTCCCTGAATGTTCGC<br>R：TCCTCATCCAGCACGTAGAAG
ST7	F：GTATCTGTGGACCGTGTGGTT<br>R：AGTTGTCGTTGATTTTCAAAGGC
TRAF7	F：TCTGCGCTCCACATTCTCAC<br>R：ACCGCGATGTTGTTCACCA
β-Actin	F：CCTGGCACCCAGCACAAT<br>R：GGGCCGGACTCGTCATAC
miR-3656	GGCGGGUGCGGGGGUGG

Table S2 | Summary of microRNAs that are significantly different between CDEs and NDEs

miRNA_ID	NF-9	NF-16	NF-24	NF-26	NF-28	TF-9	TF-16	TF-24	TF-26	TF-28
hsa-miR-3656	0.000	-0.750	0.000	-0.807	-0.289	2.543	2.283	1.808	1.945	2.370
hsa-miR-1273f	-0.502	0.029	-0.352	0.000	0.188	0.453	0.033	-0.341	0.276	0.195
hsa-miR-3196	0.043	-0.051	0.000	0.000	-0.068	0.480	0.375	0.057	0.055	0.354
hsa-miR-126-3p	-0.201	-0.750	0.810	0.554	-0.465	-0.456	0.000	0.000	0.958	0.085
hsa-miR-5096	0.000	0.551	-0.652	0.000	0.188	0.480	0.033	-0.040	0.390	0.143
hsa-miR-4492	-0.024	0.205	-0.352	0.000	0.275	0.768	0.589	-0.040	0.984	0.620
hsa-miR-3135b	0.540	-0.051	0.046	-0.029	0.079	0.726	0.902	0.773	1.206	0.893
hsa-miR-451a	-0.325	0.154	1.600	-0.108	-0.289	0.152	-0.189	0.358	-0.025	0.000
hsa-miR-4466	-0.502	0.913	0.192	0.000	0.846	0.394	0.589	0.534	0.555	0.602
hsa-miR-3960	0.344	1.029	-0.352	-0.029	0.974	0.912	0.749	0.960	0.930	0.930
hsa-miR-185-5p	0.976	0.292	1.665	-0.108	-0.465	-0.276	-0.366	-0.040	-0.121	0.143
hsa-miR-486-5p	0.198	0.481	1.943	0.147	0.012	0.328	-0.064	0.203	0.532	0.319
hsa-miR-4508	0.613	0.904	0.192	0.037	1.060	1.338	0.796	0.437	1.055	1.138
hsa-miR-4488	0.800	1.263	0.523	0.193	1.327	0.740	0.714	1.136	0.657	0.956
hsa-miR-4497	1.101	1.259	0.810	0.471	1.344	1.185	1.605	1.221	1.352	1.519
hsa-miR-4532	0.831	1.727	0.551	0.783	1.625	1.407	1.041	1.516	1.413	1.481
hsa-miR-4516	1.004	1.819	0.523	0.794	1.560	1.625	1.351	1.150	1.235	1.643
hsa-miR-342-3p	1.960	0.612	1.173	0.493	1.545	0.328	-0.666	1.238	0.122	0.655
hsa-miR-122-5p	0.660	0.841	2.303	1.837	0.836	1.068	1.112	0.889	2.315	1.026
hsa-miR-222-3p	2.082	1.076	1.301	1.184	1.438	0.550	0.825	1.717	0.180	1.003
hsa-miR-619-5p	1.147	2.107	1.029	0.948	2.098	1.904	1.725	1.522	2.111	1.927
hsa-miR-7641	2.120	1.638	1.500	2.366	1.842	0.394	0.564	-0.341	0.276	0.354
hsa-miR-92a-3p	2.723	1.441	1.993	1.650	2.125	1.290	0.825	1.877	0.889	1.330
hsa-miR-148a-3p	2.509	1.761	2.496	1.658	2.167	1.688	1.024	1.370	2.132	1.638
